# Biomechanical study of different internal fixation devices for femoral neck fractures: finite element analysis based on different reduction qualities

**DOI:** 10.3389/fbioe.2025.1678294

**Published:** 2025-09-30

**Authors:** Zhexi Zhu, Zhongjian Tang, Yafei Lu, Tao Yang, Jizhe Lu, Chenglong Pang, Bin Wang

**Affiliations:** ^1^ Department of Orthopaedics, The Second Affiliated Hospital of XuZhou Medical University, Xuzhou, China; ^2^ Graduate School of Xuzhou Medical University, Xuzhou, China; ^3^ Graduate School, Hebei Medical University, Hebei, China; ^4^ Department of Trauma Orthopaedics, Peking University People’s Hospital, Beijing, China

**Keywords:** femoral neck fracture, reduction quality, BDSF, hollow screw, finite element analysis

## Abstract

**Objective:**

To compare and analyze the biomechanical advantages and disadvantages of four cannulated screws (4CCS) internal fixation device, biplanar double support screw (BDSF) internal fixation device, and dynamic hip screw (DHS) internal fixation device in anatomical reduction, positive reduction, and negative reduction of femoral neck fractures with a Pauwels angle of 50° using the finite element method.

**Methods:**

Based on the finite element method, a femoral neck fracture model with a Pauwels angle of 50° was established. Anatomical reduction, positive reduction, and negative reduction femoral neck fracture models were respectively constructed on this basis. Then, 4CCS, BDSF, and DHS internal fixation devices were implanted into the three types of femoral neck models, resulting in a total of 9 groups of finite element models. Each fracture group was tested under an axial load of 2100 N, and the displacement of the femur and internal fixation devices, as well as the distribution of Von Mises stress (VMS), were measured.

**Results:**

In the femoral neck fracture model with a 50° Pauwels angle, maximum femoral displacement in the BDSF group was 3.412 mm for anatomical reduction, 3.459 mm for positive reduction, and 3.962 mm for negative reduction—all smaller than those in the 4CCS and DHS groups. For femoral head stress, the 4CCS group showed higher stress (42.90 Mpa) than BDSF (38.21 Mpa) and DHS (35.91 Mpa) under anatomical reduction. However, under positive and negative reduction, BDSF (23.75 Mpa and 27.9 Mpa) outperformed 4CCS (50.40 Mpa and 34.6 Mpa) and DHS (44.92 Mpa and 44.6 Mpa). Negative reduction models had significantly greater overall stress than positive reduction models, with positive reduction showing better stability. Under anatomical reduction, BDSF’s internal fixation stress (222.3 Mpa) and displacement (3.611 mm) differed notably from DHS (322.2 Mpa, 3.009 mm) and 4CCS (276.0 Mpa, 3.346 mm). Under positive and negative reduction, BDSF (247.4 Mpa/3.370 mm and 292.1 Mpa/3.865 mm) performed better than 4CCS (250.4 Mpa/3.480 mm and 293.1 Mpa/3.897 mm). BDSF and 4CCS had significantly lower internal fixation stress than DHS under positive and negative reduction, while no significant difference in internal fixation displacement was found between BDSF and DHS under these conditions.

**Conclusion:**

For femoral neck fractures with a Pauwels angle of 50° under anatomical reduction, positive reduction, and negative reduction, the BDSF internal fixation device has better biomechanical performance than the 4CCS and DHS internal fixation devices. Except for anatomical reduction, positive reduction can achieve better biomechanical results. The BDSF internal fixation technique can be considered a reliable closed reduction internal fixation technique for treating femoral neck fractures with different anatomical reductions.

## 1 Introduction

Femoral neck fractures (FNFs) are a common type of bone injury, accounting for approximately 3.6% of all fractures and as high as 50% of hip fractures (Luo et al., 2023). With the rapid development of social economy and changes in lifestyle, high-energy injuries such as car accidents and falls from heights have led to a significant increase in femoral neck fractures, especially among young adults, posing major challenges in the field of orthopedic treatment (Wan et al., 2021). In the treatment of femoral neck fractures, the quality of intraoperative reduction is considered a key factor affecting prognosis (Wan et al., 2021). Studies have shown that early anatomical reduction and effective maintenance of femoral head blood supply can significantly reduce the risk of postoperative complications such as femoral neck shortening and femoral head necrosis (Wang et al., 2019). However, due to the strict requirements of anatomical reduction, excessive pursuit of it may damage the blood supply to the femoral head and neck through repeated reduction or open reduction, which is instead detrimental to prognosis (Zhu et al., 2015). In this regard, OTFRIED proposed the classification of non-anatomical reduction: positive reduction and negative reduction, where the former refers to the proximal fragment shifting upward relative to anatomical reduction, and the latter refers to shifting downward (Gotfried et al., 2013). Studies have proven that compared with negative reduction, positive reduction exhibits better biomechanical stability in femoral neck fractures (Ding et al., 2024).

In addition, the choice of internal fixation device is also crucial for treatment outcomes (Wang et al., 2022). Currently, various internal fixation techniques such as dynamic hip screw (DHS), proximal femoral locking plates (PFLP), cannulated screws (CCS), femoral neck system (FNS), dynamic condylar screw (DCS), and percutaneous compression plate (PCCP) are widely used in the treatment of femoral neck fractures (Tang et al., 2024). Although medial buttress plates and intramedullary fixation systems have better anti-rotation and anti-shear effects than cannulated compression screws, the problems of large trauma and significant impact on blood supply cannot be ignored (Lu-Yao et al., 1994; Zlowodzki et al., 2008; Chen et al., 2017). At present, closed reduction with CCS internal fixation has become the mainstream treatment for femoral neck fractures in young adults, and the accurate positioning and fixation of CCS are directly related to clinical prognosis (Florschutz et al., 2015). With the introduction of orthopedic surgical robots, the problem of accurate positioning of CCS has been alleviated, but the biomechanical effects of different internal fixation techniques under different reduction qualities still need in-depth exploration (Thakkar et al., 2017).

For vertical femoral neck fractures, the traditional three-screw fixation method has limited efficacy, often accompanied by high rates of nonunion and femoral head necrosis. To this end, some scholars have proposed adding a horizontal cannulated lag screw to enhance shear resistance and improve fixation efficacy based on three screws (Gümüştaş et al., 2014; Li et al., 2017). Although the dynamic hip screw (DHS) can provide stable fixation, its disadvantages such as complex surgery, large trauma, and heavy bleeding cannot be ignored. The biplanar double support screw (BDSF) technique proposed by Orlin Filipov effectively distributes stress loads, enhances femoral head stability, and reduces total displacement by innovatively arranging three CLSs on two vertical planes, showing good application prospects (Filipov, 2019). Currently, three different internal fixation devices and three different reduction methods have corresponding research reports in clinical practice and have achieved certain clinical efficacy, but there is a lack of corresponding biomechanical comparative analysis. This study aims to compare the biomechanical advantages and disadvantages of three different internal fixation devices in the treatment of femoral neck fractures under three different reduction methods through biomechanical analysis, and to compare the biomechanical impacts of different internal fixation methods and different reduction methods in the treatment of femoral neck fractures.

## 2 Materials and methods

### 2.1 Establishment of finite element analysis model

A healthy male volunteer, aged 45 years, with a height of 175 cm and a weight of 78 kg, was selected. Written informed consent was obtained from the participant, explicitly authorizing the use of his imaging data for scientific research. The study protocol was reviewed and approved by the Ethics Committee of the Second Affiliated Hospital of Xuzhou Medical University. All procedures performed were in accordance with the ethical standards outlined in the Declaration of Helsinki. A Siemens 128-slice spiral CT scanner from the Second Affiliated Hospital of Xuzhou Medical University was used. Thin-slice CT scans of the volunteer’s pelvis and lower limbs were performed. The patient’s imaging data were read and saved in DICOM format.

**FIGURE 1:**
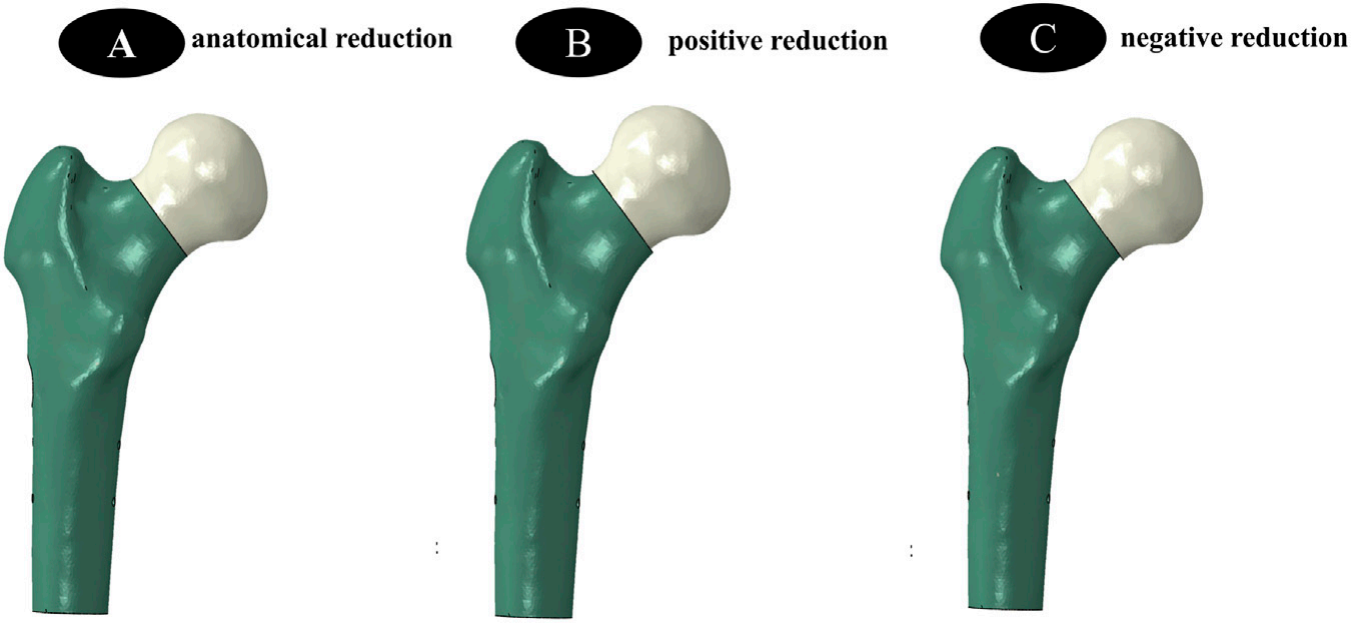
**(A–C)** show three different reduction methods, namely the establishment of internal fixation models for anatomical reduction, positive reduction, and negative reduction, respectively.

The DICOM format was imported into Mimics 20.0 software. The STL file of the femoral model exported from Mimics was imported into Geomagic, where surface defect repair was performed using the mesh derivation function to close the model. Then, the complex surface model of the femur was reconstructed through polygon and non-uniform rational B-spline (NURBS) surface fitting and mesh relaxation, and each segment model was exported in *.iges format. The above surface model was imported into ProEngineer 5.0 software (PTC, the United States) for smoothing, meshing, noise reduction, and surface adaptation to establish a three-dimensional solid model, and then imported into SolidWorks 2017 software (Dassault, France). Boolean operations were used to establish three-dimensional models of cortical bone and cancellous bone, and a femoral neck fracture model with a Pauwels angle of 50° was constructed. Based on anatomical reduction, the proximal femur was translated 2 mm obliquely upward to obtain the positive buttress model, and conversely, the negative buttress model was obtained (Figure 1). According to clinical fixation procedures and engineering geometric data, models of BDSF internal fixation device, DHS internal fixation device, and 4 cannulated screws (4CCS) internal fixation device were generated using SolidWorks software.

### 2.2 Establishment of Internal Fixation Device Models

**FIGURE 2:**
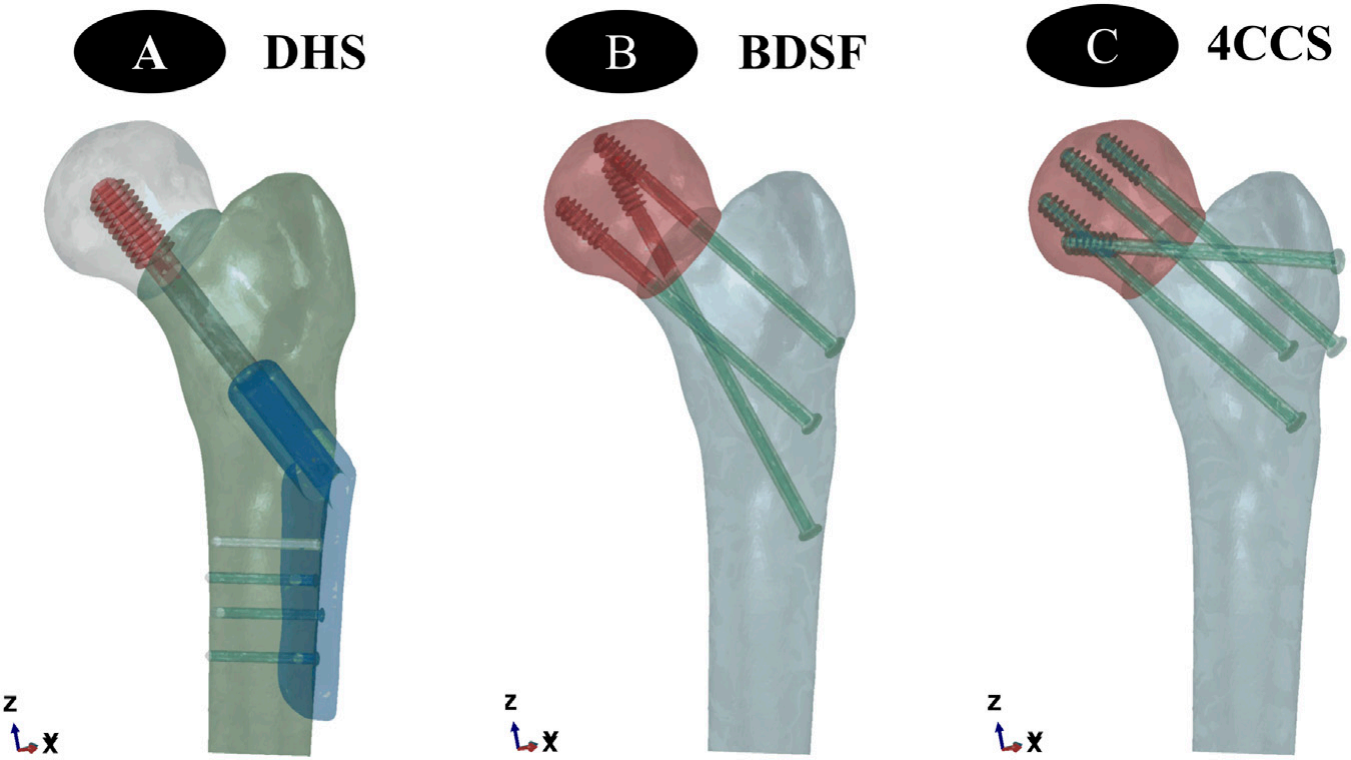
**(A–C)** show the establishment of three different internal fixation methods, namely DHS, BDSF, and 4CCS internal fixation methods, respectively.

BDSF Internal Fixation Device Model: The distal screw should be close to the calcar femorale, forming a 165° angle with the femoral shaft. In the lateral view, the entry points of the three screws are at the posterior 1/3 of the lateral femoral cortex. The entry point of the middle screw is 4 cm below the greater trochanter, and the entry point of the proximal screw is 2 cm above the middle screw. In the lateral view, the proximal and middle screws are located in the anterior 1/3 of the femoral head, entering perpendicular to the fracture line. The distal ends of the 3 CCSs are approximately 5 mm from the articular surface (Figure 2).

DHS Internal Fixation Device Model:The dynamic hip screw was inserted into the central position of the femoral head and neck, with the lateral plate closely attached to the femoral cortex, and the tip-apex distance of the screw in the femoral head was 5 mm.

4CCS Internal Fixation Device Model:Two cannulated compression screws were inserted into the femoral head from a position approximately 2 cm below the top of the greater trochanter, close to the femoral neck cortex. Another cannulated compression screw was inserted into the femoral head from a position approximately 4 cm below, close to the calcar femorale, so that the 3 screws were distributed in a close-to-edge, parallel, inverted triangular pattern, with the screw tips 0.5–1.0 cm below the femoral head cartilage surface. After fixing the 3 cannulated compression screws, an additional cannulated compression screw was inserted near the greater trochanter, approximately perpendicular to the fracture line.

### 2.3 Material parameter setting

All experimental models were ideal continuous, uniform, isotropic linear elastic materials. According to previous studies (Taylor et al., 1996), the material parameters of the finite element models in this study are shown in the table (Table 1).

**TABLE 1:** Material parameters of the finite element model.

Materials	(MPa)	Poisson’s ratio
Cortical bone	16,800.0	0.3
Cancellous bone	840.0	0.3
Head of femur	900.0	0.29
Collum femoris	620.0	0.29
Titanium alloy	110,000.0	0.3

### 2.4 Boundary conditions and loads

The force on the femur is complex. During daily movements such as gait, the maximum load borne by the hip joint can reach 2.6–4.1 times the body weight, and it increases with faster walking speed, longer step length, or increased body weight. The muscular forces acting on the femur are highly complex. Taylor et al. (1996) pointed out that introducing muscle loads into the femoral model involves multiple uncertainties, including the selection of muscle quantity, the influence of gravity, and the direction of muscle force application. Particularly in dynamic simulation scenarios, achieving accurate modeling is extremely challenging. To simplify the analysis process and focus on the fixation effects of the two groups of models, this study, referring to relevant literature (Cui et al., 2020; Wang et al., 2020; Mei et al., 2014), applied a vertical force of 2100 N to the femoral head to simulate physiological states such as bipedal standing, single-leg support, and stair climbing in humans. Full constraint treatment was applied to all nodes below the distal condyle of the femur.

### 2.5 Validity verification

To verify the validity of the models, a complete femoral model was first constructed, and material properties were assigned according to the methods described in the literature (Tsun et al., 2020; Papini et al., 2007). Under the conditions of full constraint at the lower end of the model and a vertical load of 2100 N applied to the femoral head, Ansys19.0 software (ANSYS, United States) was used for analysis, and the results were compared and verified with the reported data in the literature (Tsun et al., 2020; Papini et al., 2007).

### 2.6 Evaluation metrics

Simulation calculations were performed using Ansys19.0 software. The core comparison indicators were the distribution characteristics of maximum Von Mises stress (VMS) and maximum deformation of the two groups of models under a 2100 N load.

## 3 Results

### 3.1 Validity verification results

Under a pure compressive load of 2100 N, the compressive stiffness of the complete model was 0.798 kN/mm, which was highly consistent with the compressive stiffness [(0.76 ± 0.26) kN/mm] reported in the literature (Tsun et al., 2020; Papini et al., 2007). The stress distribution in the femur showed a gradual increasing trend from the distal end to the proximal end, with the highest stress peak located in the femoral neck. This characteristic was consistent with the research conclusions of Fernandez et al. (2013). Considering the individual differences among models, the models constructed in this experiment were deemed valid.

### 3.2 Von Mises stress (VMS) distribution and results of three CCS internal fixation devices and femur

**FIGURE 3:**
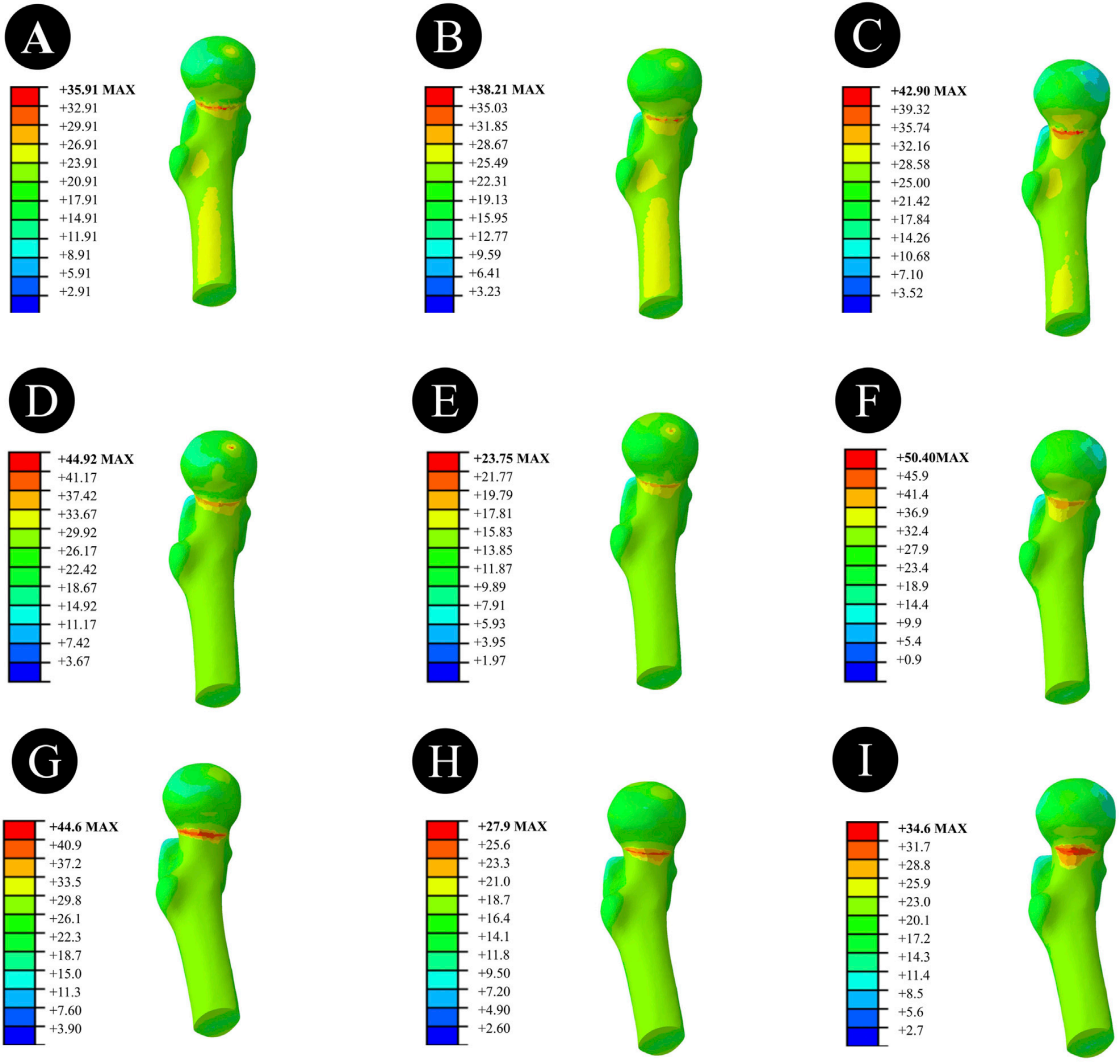
Overall femoral stress of different internal fixation methods under three different reduction methods. **(A,D,G)** Overall femoral stress of DHS under anatomical reduction, positive reduction, and negative reduction. **(B,E,H)** Overall femoral stress of BDSF under anatomical reduction, positive reduction, and negative reduction. **(C,F,I)** Overall femoral stress of 4CCS under anatomical reduction, positive reduction, and negative reduction.

**FIGURE 4:**
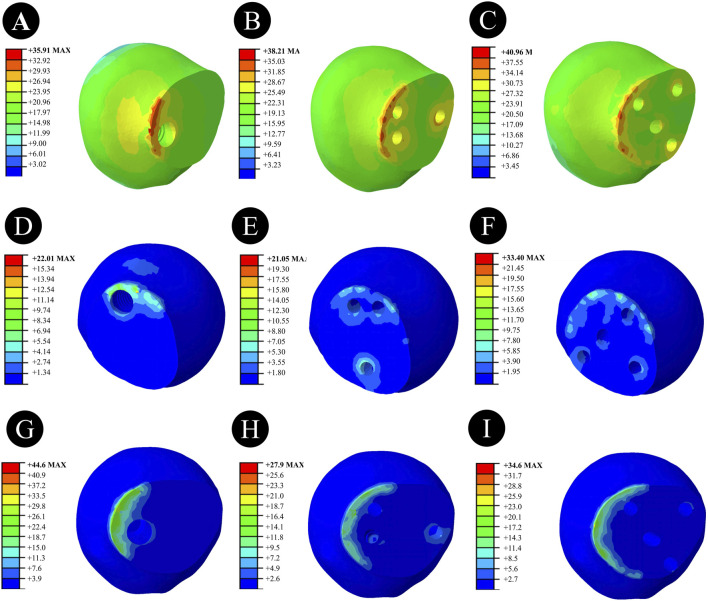
Femoral head stress of different internal fixation methods under three different reduction methods. **(A,D,G)** Femoral head stress of DHS under anatomical reduction, positive reduction, and negative reduction. **(B,E,H)** Femoral head stress of BDSF under anatomical reduction, positive reduction, and negative reduction. **(C,F,I)** Femoral head stress of 4CCS under anatomical reduction, positive reduction, and negative reduction.

**FIGURE 5:**
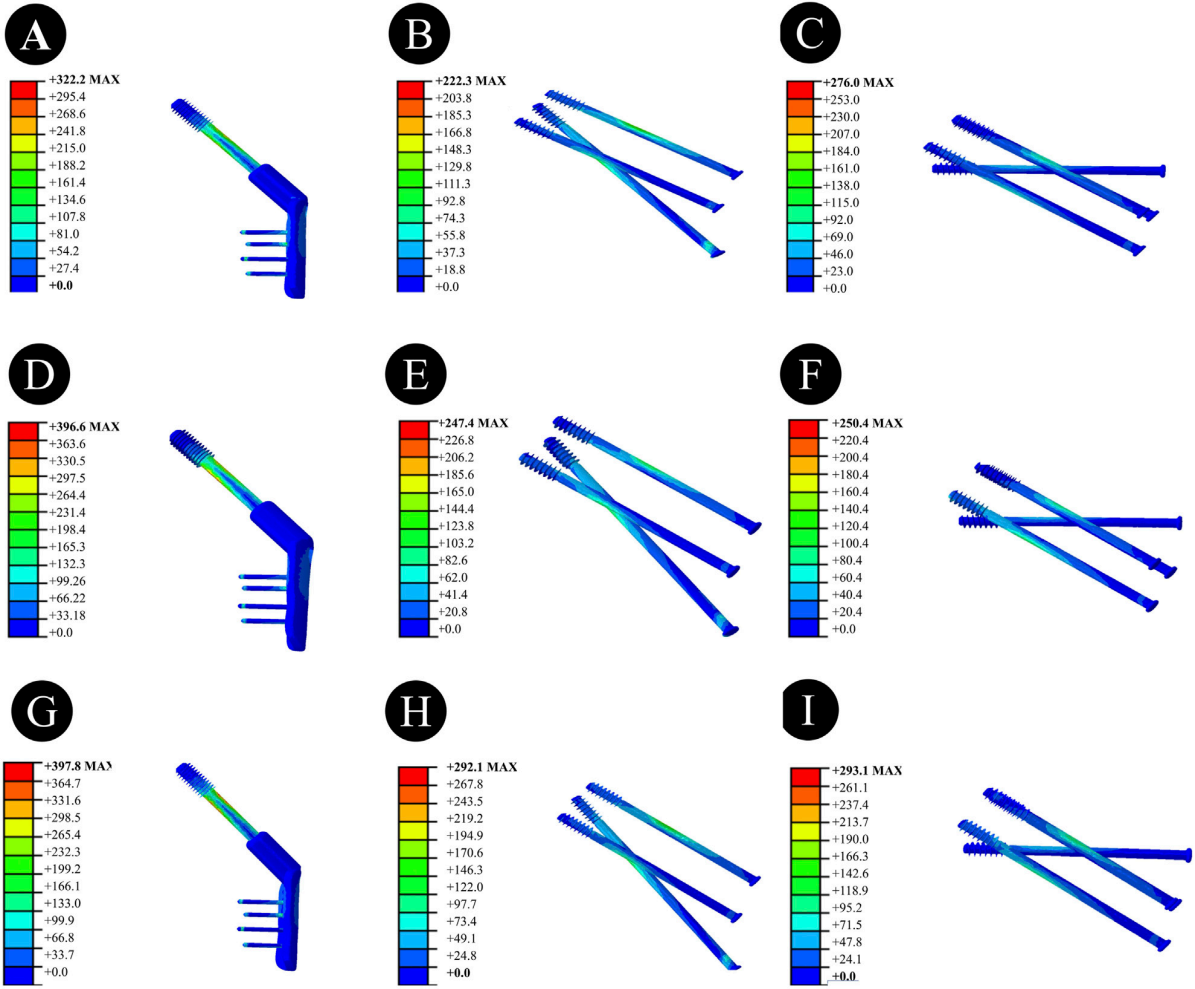
Stress of internal fixation devices with different internal fixation methods under three different reduction methods. **(A,D,G)** Stress of the internal fixation device for DHS under anatomical reduction, positive reduction, and negative reduction. **(B,E,H)** Stress of the internal fixation device for BDSF under anatomical reduction, positive reduction, and negative reduction. **(C,F,I)** Stress of the internal fixation device for 4CCS under anatomical reduction, positive reduction, and negative reduction.

Anatomically Reduced FNFs:The peak VMS of the femur in the DHS group was 35.91 MPa, that in the BDSF group was 38.21 MPa, and that in the 4CCS group was 42.90 MPa. The peak VMS of the DHS internal fixation device was 322.2 MPa, that of the BDSF internal fixation device was 222.3 MPa, and that of the 4CCS internal fixation device was 276.0 MPa. According to the displacement profile of Pauwels fractures in 50° FNFs, the VMS of the femur occurred in the cortical area below the fracture, and the VMS of the internal fixation device was concentrated on the screw surface near the fracture line (Figures 3-5).

In Pauwels type femoral neck fractures (FNFs) with a 50° angle, the peak Von Mises stress (VMS) values of the femur and internal fixation devices are as follows: For the femur, the peak VMS of DHS is 44.92 MPa, that of BDSF is 23.75 MPa, and that of 4 cannulated screws (4CCS) is 50.4 MPa. For the internal fixation devices, the peak VMS of DHS is 396.6 MPa, that of BDSF is 247.4 MPa, and that of 4CCS is 250.4 MPa. According to the displacement profile of such fractures, the VMS of the femur occurs in the cortical area below the fracture, while the VMS of the internal fixation devices is concentrated on the surface of the screws near the fracture line.

Negatively Reduced FNFs:The peak VMS of the femur in the DHS group was 44.6 MPa, that in the BDSF group was 27.9 MPa, and that in the 4CCS group was 34.6 MPa. The peak VMS of the DHS internal fixation device was 397.8 MPa, that of the BDSF internal fixation device was 292.1 MPa, and that of the 4CCS internal fixation device was 293.1 MPa. According to the displacement profile of Pauwels fractures in 50° FNFs, the VMS of the femur occurred in the cortical area below the fracture, and the VMS of the internal fixation device was concentrated on the screw surface near the fracture line.

### 3.3 Displacement results and distribution of three CCS internal fixation devices and femur

**FIGURE 6:**
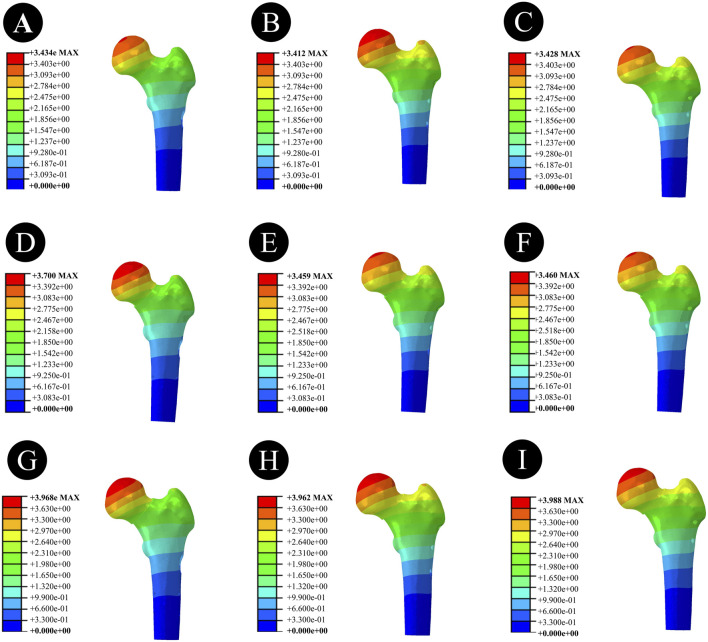
Overall femoral displacement of different internal fixation methods under three different reduction methods. **(A,D,G)** Overall femoral displacement of DHS under anatomical reduction, positive reduction, and negative reduction. **(B,E,H)** Overall femoral displacement of BDSF under anatomical reduction, positive reduction, and negative reduction. **(C,F,I)** Overall femoral displacement of 4CCS under anatomical reduction, positive reduction, and negative reduction.

**FIGURE 7:**
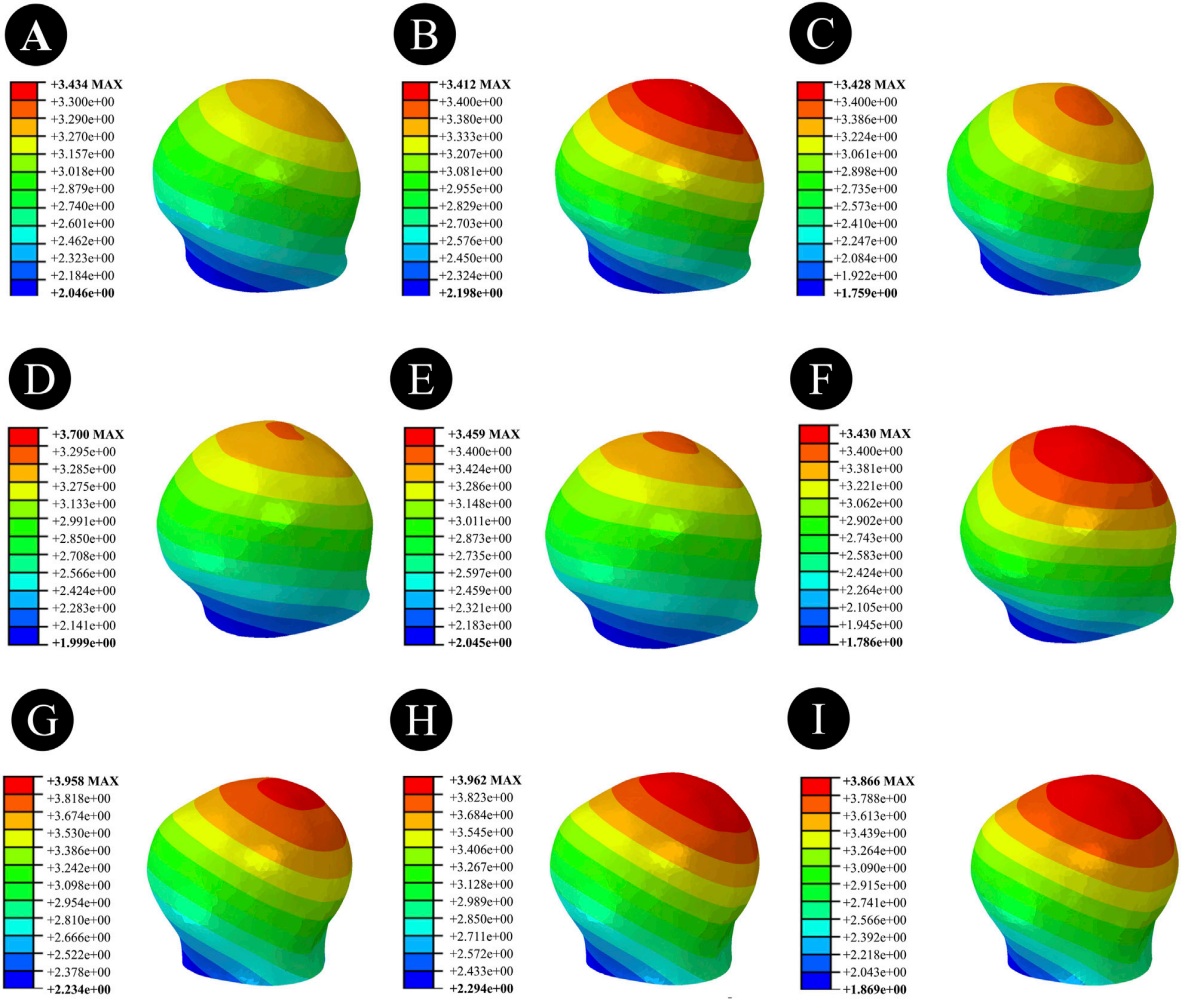
Femoral head displacement of different internal fixation methods under three different reduction methods. **(A,D,G)** Femoral head displacement of DHS under anatomical reduction, positive reduction, and negative reduction. **(B,E,H)** Femoral head displacement of BDSF under anatomical reduction, positive reduction, and negative reduction. **(C,F,I)** Femoral head displacement of 4CCS under anatomical reduction, positive reduction, and negative reduction.

**FIGURE 8:**
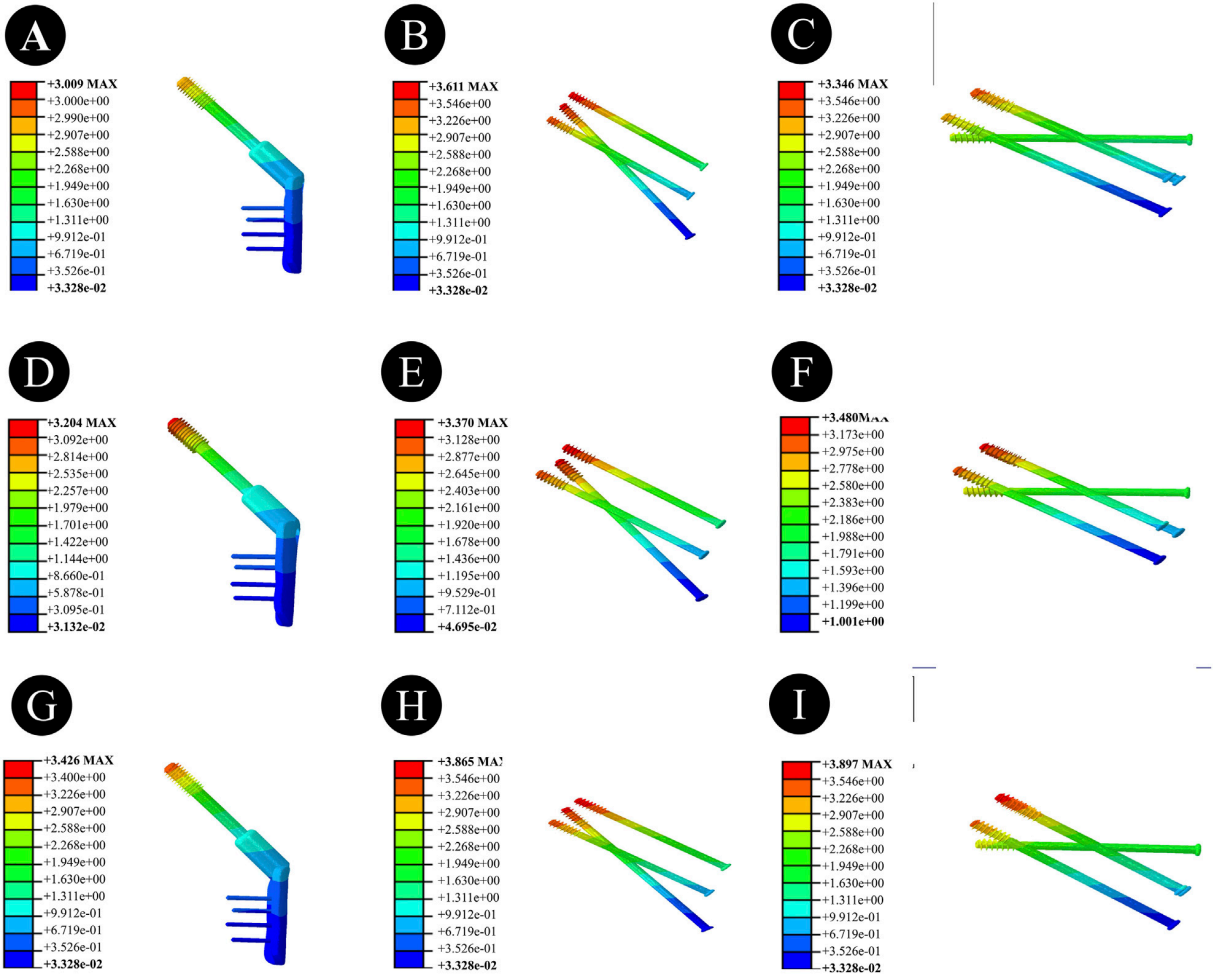
Displacement of internal fixation devices with different internal fixation methods under three different reduction methods. **(A,D,G)** Displacement of the internal fixation device for DHS under anatomical reduction, positive reduction, and negative reduction. **(B,E,H)** Displacement of the internal fixation device for BDSF under anatomical reduction, positive reduction, and negative reduction. **(C,F,I)** Displacement of the internal fixation device for 4CCS under anatomical reduction, positive reduction, and negative reduction.

Anatomically Reduced FNFs:The maximum femoral displacement in the DHS group was 3.434 mm, that in the BDSF group was 3.412 mm, and that in the 4CCS group was 3.428 mm. The maximum displacement of the DHS internal fixation device was 3.009 mm, that of the BDSF internal fixation device was 3.611 mm, and that of the 4CCS internal fixation device was 3.346 mm. According to the displacement profile of Pauwels fractures in 50° FNFs, the maximum displacement occurred in the upper part of the femoral head, and the maximum displacement of the internal fixation device occurred at the top of the screw (Figures 6–8).

Positively Reduced FNFs:The maximum femoral displacement in the DHS group was 3.700 mm, that in the BDSF group was 3.459 mm, and that in the 4CCS group was 3.460 mm. The maximum displacement of the DHS internal fixation device was 3.204 mm, that of the BDSF internal fixation device was 3.370 mm, and that of the 4CCS internal fixation device was 3.480 mm. According to the displacement profile of Pauwels fractures in 50° FNFs, the maximum displacement occurred in the upper part of the femoral head, and the maximum displacement of the internal fixation device occurred at the top of the screw.

Negatively Reduced FNFs:The maximum femoral displacement in the DHS group was 3.968 mm, that in the BDSF group was 3.962 mm, and that in the 4CCS group was 3.988 mm. The maximum displacement of the DHS internal fixation device was 3.426 mm, that of the BDSF internal fixation device was 3.865 mm, and that of the 4CCS internal fixation device was 3.897 mm. According to the displacement profile of Pauwels fractures in 50° FNFs, the maximum displacement occurred in the upper part of the femoral head, and the maximum displacement of the internal fixation device occurred at the top of the screw.

### 3.4 Distribution patterns of displacement and stress under three different reduction techniques with various internal fixation methods

Figure 9.

**FIGURE 9:**
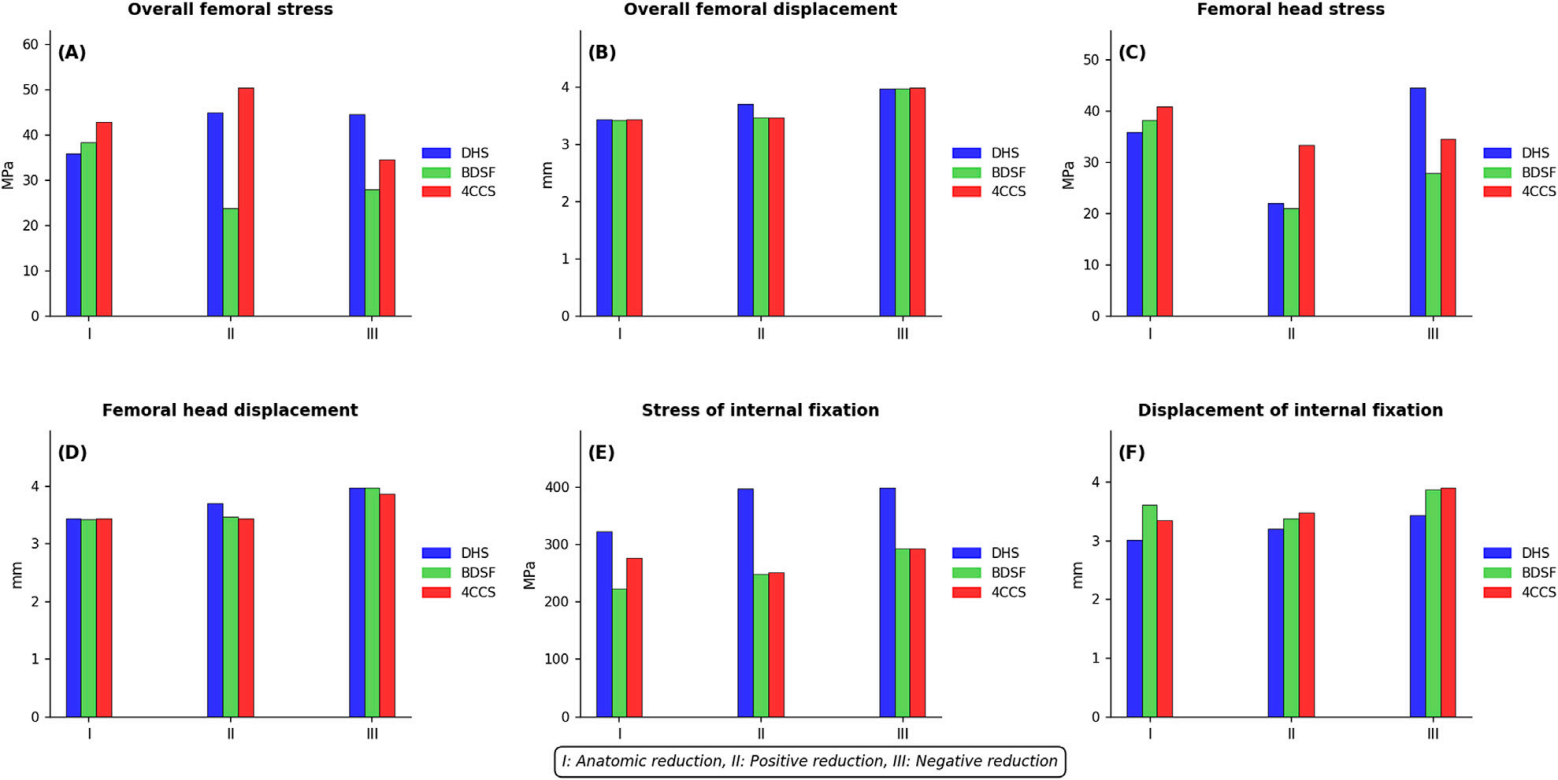
Distribution Patterns of Displacement and Stress under Three Different Reduction Techniques with Various Internal Fixation Methods: **(A)** Overall Femoral von Mises Stress **(B)** Overall Femoral Displacement **(C)** Femoral Head von Mises Stress **(D)** Femoral Head Displacement **(E)** Internal Fixation von Mises Stress **(F)** Internal Fixation Displacement.

## 4 Discussion

Femoral neck fractures in young adults are mostly caused by high-energy injuries, which often result in vertical femoral neck fractures (Wan et al., 2021; Al-Ani et al., 2015). Relevant studies have shown that in vertical fractures, shear and rotational forces can cause oscillation and rotation of the femoral head, easily increasing the risk of postoperative complications such as malunion and avascular necrosis of the femoral head (Wan et al., 2021). Therefore, the treatment of vertical femoral neck fractures has always been a focus of clinical attention. In recent years, with the popularization of computing technology, finite element analysis has become a widely used stress analysis method in biomechanical research. By constructing biomechanical finite element models, repeated analyses can be conducted without violating ethical principles, and the results can provide a reliable basis for the selection of internal fixation methods and reduction methods for vertical femoral neck fractures.

For patients with vertical femoral neck fractures, the key to treatment lies in protecting the blood supply to the femoral head, ensuring good reduction quality, and selecting a firm fixation method. Studies have found that reducing the number of reductions and achieving closed reduction are effective measures to protect blood supply. Clinically, anatomical reduction should be pursued as much as possible, but in practice, anatomical reduction is difficult to achieve. Forced pursuit may damage the blood supply to the femoral head, leading to a series of adverse complications (Wan et al., 2021). Israeli doctor Yechiel Gotfried classified non-anatomical reduction into positive reduction and negative reduction. Studies have confirmed that the surgical failure rate of negative reduction is higher than that of positive reduction, so positive reduction is advocated as the priority (Gotfried et al., 2013). Zhou xiang et al. established an intramedullary nail fixation model through finite element analysis and found that the maximum stress of positive reduction internal fixation was less than that of negative reduction internal fixation, believing that positive reduction has better biomechanical stability (Zhou et al., 2024). Huang et al. inferred that in the positive reduction state, the proximal fracture fragment is supported12 by the medial cortex of the femoral neck, which can convert shear force into a force that promotes healing of the lower part of the fracture line (Huang et al., 2020).

The three internal fixation models constructed in this study also drew similar conclusions in positive and negative reductions. The maximum internal fixation stresses of DHS, BDSF, and 4CCS under positive reduction were 396.6 Mpa, 247.4 Mpa, and 250.4 Mpa, respectively, and those under negative reduction were 397.8 Mpa, 292.1 Mpa, and 293.1 Mpa, respectively. This indicates that positive reduction is significantly better than negative reduction. This result is consistent with previous studies, that is, positive reduction is the preferred option when anatomical reduction cannot be achieved, and negative reduction should be avoided (Gotfried et al., 2013; Zhou et al., 2024; Huang et al., 2020).

At present, three cannulated screws (3CCS) and DHS are the more commonly used internal fixation methods for femoral neck fractures in young adults (Stoffel et al., 2017). 3CCS has the advantages of small trauma and short operation time. It adopts a fixation method parallel to the calcar femorale, which is more suitable for patients with non-vertical fractures. However, studies have reported that in vertical fractures, treatment with 3CCS is prone to nonunion and internal fixation device fracture (Johnson et al., 2017; Xia et al., 2021). To this end, some scholars have proposed adding an additional cannulated compression screw near the greater trochanter, approximately perpendicular to the fracture line, on the basis of 3CCS to enhance shear resistance and improve fixation efficacy (Gümüştaş et al., 2014; Li et al., 2017). Moreover, the stability and clinical efficacy of four cannulated screws (CCS) fixation are better than those of three cannulated screws (Gümüştaş et al., 2014). Although dynamic hip screw (DHS) can provide strong fixation for femoral neck fractures with good early treatment efficacy, it is associated with complex surgery, large trauma, and relatively more intraoperative blood loss (Magone et al., 2018; Tianye et al., 2019).

The biplanar double support screw (BDSF) internal fixation technique is a new type of internal fixation technique proposed by Orlin Filipov (Filipov, 2019), which places 3 CCSs on two vertical planes. Among them, the distal screw is located on the dorsal plane, and the middle and proximal screws are located on the ventral plane. The distal screw, as the main support screw, has support points on the distal cortex of the femoral neck and the lateral cortex of the femoral shaft, and provides additional support for the posterior cortex of the femoral neck. This biplanar double support structure can resist shear and rotational stresses by bearing axial compressive stress. Studies have shown that compared with the traditional multiple parallel CCS internal fixation technique, BDSF can provide additional effective support for the posteroinferior cortex of the femoral neck, improving the overall stability and shear resistance of the internal fixation device (Tang et al., 2024; Liporace et al., 2008; Ye et al., 2015).

The results of this study are somewhat different from previous studies. In the anatomically reduced fracture model, the maximum stress of the femoral head and the maximum stress of the internal fixation device in the 4CCS internal fixation group were significantly less than those in the BDSF and DHS groups, but the maximum displacements of the femoral head in the 4CCS and DHS groups were higher than those in the BDSF group. In positive buttress reduction, the maximum stress of the femoral head in the BDSF group was significantly less than that in the 4CCS and DHS groups, and the displacement of the internal fixation device was significantly better than that in the 4CCS group, with no significant difference from the DHS group. In the anatomical reduction state, the stress and displacement of the internal fixation device in the BDSF group differed greatly from those in the DHS and 4CCS groups. In positive buttress and negative buttress reductions, the BDSF group performed better than the 4CCS group, and the internal fixation stresses of both groups were significantly better than that of the DHS group, while there was no significant difference in internal fixation displacement between the BDSF group and the DHS group. It is noteworthy that under conditions of anatomic reduction, the Dynamic Hip Screw (DHS) exhibits a modest superiority over the Bi-plane Double Support Fixation (BDSF) in terms of femoral head stress (35.91 MPa vs. 38.21 MPa) and implant displacement (3.009 mm vs. 3.611 mm). This advantage may be attributed to the structural characteristics of the DHS, such as its side plate design, which allows more effective stress dispersion under axial loading when reduction is ideal. However, the clinical significance of the BDSF lies in its bi-plane double-support structure, which provides enhanced resistance against shear and rotational stresses—particularly in non-anatomic reductions (e.g., positive reduction). Such scenarios are more frequently encountered in clinical practice. Thus, while the DHS demonstrates slightly superior biomechanical performance under ideal anatomic conditions, the BDSF offers more comprehensive mechanical advantages in the more prevalent non-ideal reduction settings.

In summary, the results of this study show that the maximum stress and displacement of the femoral head, as well as the maximum stress and displacement of the internal fixation device in negative reduction, are significantly greater than those in positive reduction, and anatomical reduction is better than positive reduction. Therefore, patients with femoral neck fractures should strive for anatomical reduction as much as possible, and if anatomical reduction cannot be achieved, positive reduction is advocated, while negative reduction should be avoided. This study clarifies the advantages and disadvantages of three different internal fixation devices under different reduction qualities, which can provide guidance for the selection of surgical methods and fracture reduction methods.

This study has certain limitations. ① The Pauwels angle of femoral neck fractures was only 50°, not covering other angle ranges, and the selection of fracture reduction methods and internal fixation devices was limited to fractures at specific angles, so the results cannot support the application of other fracture types and internal fixation devices. ② The material parameters of the model used in the study were set as isotropic elastic materials, which differ from the anisotropic material properties of actual human bones. Although this study mainly modeled the overall trend, which is reasonable to a certain extent, the results still need to be interpreted with caution. ③ This study applied only a 2100 N vertical axial compressive load to simulate single-leg stance and did not account for the influence of muscular forces, such as those from the abductor muscles. This simplification may lead to an overestimation of displacement and stress values in all internal fixation models, particularly under malreduced conditions. Although such simplifications are common in finite element studies and help control variables, future research should incorporate multidirectional loading and muscle force simulations to more accurately represent biomechanical behavior under physiological conditions.④This study did not account for variations in bone quality, such as osteoporosis, which may constrain the generalizability of the findings in clinical settings. Future research should build upon this work by incorporating bone density gradients to further evaluate the biomechanical performance of various internal fixation methods under osteoporotic conditions.

## Data Availability

The raw data supporting the conclusions of this article will be made available by the authors, without undue reservation.
